# Approach to external cephalic version through social media: experience from a tertiary center in Brazil

**DOI:** 10.1590/1806-9282.20240935

**Published:** 2025-05-02

**Authors:** Suellyn de Pinho Alves, Rafaela Tessaro de Assis, Cintia Yurie Yamachi, Jorge Francisco Kuhn dos Santos, Edward Araujo, Sue Yazaki Sun

**Affiliations:** 1 Universidade Federal de São Paulo, Escola Paulista de Medicina, Department of Obstetrics – São Paulo (SP), Brazil.

**Keywords:** External cephalic version, Social media, Questionnaire

## Abstract

**OBJECTIVE:**

The aim of this study was to develop a communication and health education page on external cephalic version through the social media Instagram and, second, to assess the degree of patients’ prior understanding of the external cephalic version procedure.

**METHODS:**

We conducted a prospective cohort study on 133 singleton pregnancies (until 41 weeks of gestation), for which an online questionnaire (Google Forms) was applied on the social media Instagram, with four sections. The questionnaire was applied between January 2022 and December 2023. Before the intervention (reading the content displayed on Instagram), sections 1, 2, and 3 were applied, and after reading, section 4. Pregnant women of any gestational age who received prenatal care at our service were included. Patients<14 years of age and those who could not read were excluded.

**RESULTS:**

A statistically significant difference was found after reading the content made available on Instagram, with a positive evolution after reading (p<0.001). Of this sample, 100% felt that knowledge about the external cephalic version should reach the major population of pregnant women, 131 (98.50%) opined that they would talk to other pregnant women about it, and 113 (84.96%) said that they would perform the procedure if they had indications for it.

**CONCLUSION:**

This study provided a comprehensive overview of pregnant women's knowledge about external cephalic version. Through the results obtained, it is possible to observe the evolution of pregnant women's knowledge about external cephalic version after reading the content available on Instagram. In addition, the pregnant women expressed a strong desire to share information about the external cephalic version with other pregnant women.

## INTRODUCTION

The Internet is now the primary source of health information for people around the world, with social media increasingly serving as a channel for health education. In addition, the number of health-focused social media accounts has skyrocketed, with daily growth rates of up to 28% by 2020^1^. One of the great benefits of social media for health communication is the accessibility and reach of health information to diverse populations, regardless of age, education, race or ethnicity, and location, compared to traditional communication methods^2^.

According to the Brazilian Internet Steering Committee, 83% of Brazilian households had access to the Internet in 2020, and in absolute terms, the country now has 61.8 million connected households^3^. The social media Instagram has surpassed more than 100 million users in Brazil and involves the publication of images and videos combined with texts, enabling interaction among users and the multiplication of knowledge^4^.

Unfortunately, social media tools remain informal and unregulated mechanisms for collecting, sharing, and promoting information, so the information shared is of variable quality and consistency^2^. It is important that trained health professionals teach patients how to search and filter information on the Internet so that they do not consume low-quality content and that the information comes from reliable sources and is always confirmed in consultation with the doctor responsible for their care. One study found that the more specific a search term on the Internet, the more useful the information^5^.

External cephalic version (ECV) is a procedure performed to change the non-cephalic fetal position to a cephalic presentation^6^. In this procedure, the fetus is manipulated by applying pressure through the mother's abdominal wall to change the breech presentation to a cephalic presentation. The aim of the ECV is to increase the number of cephalic presentations and thus reduce the incidence of breech deliveries and cesarean sections, which have a higher risk of complications compared to cephalic deliveries^7^. The ECV should be performed in pregnant women>37 weeks of gestation, as the spontaneous version is likely to have already occurred and there is less chance for reversal of the ECV^8^.

Pelvic presentation occurs in 3–4% of term pregnancies^9^, and the cesarean section rate is nearly 100%^10^. This rate could be halved if ECV were performed on all eligible pregnant women, as the success rate of ECV varies between 40 and 50%^9^. However, due to a lack of public awareness of ECV and a certain reluctance on the part of physicians, this procedure is still rarely performed in Brazil.

Having in mind that social media has been commenting a lot about this procedure, especially nowadays with the increased demand for vaginal deliveries, but we have seen few healthcare physicians speaking out, it is important to share quality information with those who might benefit from it: pregnant women.

Therefore, the general objective of this study was to develop a communication and health education page on ECV through the social media Instagram, so pregnant women have knowledge of it as an option in the birth-planning process.

## METHODS

A prospective cohort study was carried out on singleton pregnant women who underwent prenatal care at the Department of Obstetrics, Paulista School of Medicine – Federal University of São Paulo (EPM-UNIFESP). It was a convenience sample, with the sample size calculated through G*power software 3.1.9.6 version, taking into consideration a low Cohen's d effect value (0.2), 5% significance level, and 80% test power.

We excluded pregnant women under the age of 14 years and illiterate women. This study was approved by the Ethics Committee of UNIFESP (CAAE: 58959922.4.0000.5505), and all participants signed the consent form.

The pregnant women were paired, and they were evaluated at time 1, before reading the content on Instagram on the website of the Department of Obstetrics of the EPM-UNIFESP about the ECV, and at time 2, after reading, to check the evolution of knowledge on the topic after reading; in other words, the progress they were able to achieve from the content shared and adherence to the procedure. Data were collected in person through an anonymous questionnaire via Google Forms (available at: https://forms.gle/5Z5uAs8AWDJkqPvt9) that included qualitative variables divided into four sections. Section 1 contained seven questions about the socioeconomic profile of pregnant women. Section 2 contained 13 questions about the current and previous pregnancies. Sections 3 and 4 had the same four questions of basic level related to the ECV, i.e., important points for lay knowledge. Section 4 had an additional six questions about the level of satisfaction with the information provided. The pregnant women were given between 5 and 7 min to complete the questionnaire.

Before the intervention (reading the content displayed on Instagram on the website of the Department of Obstetrics of the EPM-UNIFESP), Sections 1, 2, and 3 of the questionnaires were applied. After the intervention, Section 4 of the questionnaire was applied. There were seven posts for Instagram. After reading the Patient Education Materials Assessment Tool for Audiovisual Materials (PEMAT-A/V), the content to be displayed on Instagram was developed taking into account the aspects assessed by the tool. The PEMAT-A/V was developed by the Agency for Healthcare Research and Quality as a systematic method for analyzing the level of usefulness and understandability of audio–visual materials developed for patients.

The questions in Sections 1 and 2, which are qualitative variables, were compared using chi-square, Fisher's exact, or likelihood ratio test, as appropriate. The questions in Sections 3 and 4 of the questionnaires were scored, with each question worth 1 point. The numerical variables were compared using the Wilcoxon signed-rank test at a 5% significance level. A positive association was considered to exist between the intervention and a higher number of correct answers if the pregnant women had more than 75% correct answers in Section 4 or a 50% increase in the number of correct answers from Sections 3 to 4. Analyses were performed with R software version 4.0.2.

## RESULTS

In this study, 133 pregnant women were selected after the exclusion criteria. The prevalent profile of the participants was age between 30 and 39 years (n=60; 45.11%), mixed ethnicity (n=67; 50.38%), not in a stable union (n=106; 79.70%), and completed high school education (n=62; 46.62%). Most of the pregnant women had a history of ≥3 pregnancies (n=66; 49.62%), no previous delivery (n=47; 35.34%), and no history of miscarriages (n=90; 67.67%). The majority were between 27 and 41 weeks of gestation (n=69; 51.88%), had started prenatal care ≤12 weeks of gestation (n=114; 85.71%), and had high-risk pregnancies (n=105; 78.95%). Most of the participants desired a vaginal delivery (n=82; 61.65%) (Table 1).

**Table 1:** Sociodemographic characteristics of the pregnant women.

Characteristics		Participants (n)	Percentage (%)
Age (years)			
	16–19	5	3.76
	20–29	52	39.10
	30–39	60	45.11
	>40	16	12.03
Ethnicity/race			
	Asian	1	0.75
	White	46	34.59
	Mixed	67	50.38
	Black	19	14.29
Marital status			
Stable union		27	20.30
No stable union		106	79.70
Education level			
	Illiterate	1	0.75
	Incomplete primary education	6	4.51
	Complete primary education	9	6.77
	High school incomplete	15	11.28
	High school completed	62	46.62
	Higher education incomplete	12	9.02
	Higher education completed	28	21.05
Number of pregnancies			
	1	41	30.83
	2	26	19.55
	≥3	66	49.62
Number of deliveries			
	0	47	35.34
	1	36	27.07
	2	31	23.31
	≥3	19	14.29
Miscarriages			
	Yes	43	32.33
	No	90	67.67
Gestational age (weeks)			
	≤13	19	14.29
	14–26	45	33.83
	27–41	69	51.88
Classification of prenatal care			
	Low risk	28	21.05
	High risk	105	78.95
Start of prenatal care (weeks)			
	≤12	114	85.71
	13–25	16	12.03
	26–32	2	1.50
	≥33	1	0.75
Desired type of delivery			
	Vaginal delivery	82	61.65
	Cesarean section	51	38.35
	Total	133	100.00

When evaluating the evolution of the participants’ knowledge of the ECV, a statistically significant difference was identified after reading the content made available on Instagram, with a positive evolution after reading (p<0.001) and, therefore more correct answers after reading the content (Table 2).

**Table 2:** Scores before reading and after reading and participants’ progress.

	Score			p-value*
	Before reading	After reading	Evolution	
Mean	1.805	2.820	1.015	<0.001
Standard deviation	0.899	0.968	1.066	
Minimum	0	1	−1	
Maximum	4	4	4	
Median	2	3	1	

* Wilcoxon test.

An individual analysis of the answers was also carried out according to the questionnaire. In question 1, about the correct interpretation of what a pelvic presentation would be, 34.59% of the participants (n=46) got the question wrong before reading the content and got it right after reading the content (p<0.001). The same behavioral pattern was observed when analyzing the result of question 2, about the position in which it would be possible to offer the ECV to a pregnant woman, for which 32.33% of the participants (n=43) got the question wrong before reading the content and got it right after reading it (p<0.001). When asked about the purpose of the ECV, 31.58% of the participants (n=42) got it wrong initially but got it right after reading the content (p<0.001). Finally, when asked about the indications and contraindications of the procedure, 33.84% of the participants (n=45) got it wrong on the first attempt and got it right after reading the content (p<0.001) (Table 3).

**Table 3:** Individual analysis of the answers according to the questions.

Answer before and after reading the content	How does the baby look when we talk about the breech presentation?	When can the ECV be offered to pregnant women?	What is the ECV for?	Which pregnant women can have the ECV?
Kept incorrect answer	39 (29.42)§	37 (27.82)	10 (7.52)	30 (22.5)
Correct answer to incorrect	14 (10.53)	17 (12.78)	5 (3.76)	5 (3.76)
Incorrect answer to correct	46 (34.59)	43 (32.33)	42 (31.58)	45 (33.84)
Kept correct answer	34 (25.56)	36 (27.07)	76 (57.14)	53 (39.85)
p-value*	<0.001	<0.001	<0.001	<0.001

* McNemar test.

§ Mean (standard deviation).

ECV: external cephalic version.

The analysis of the trend showed that there was no statistically significant difference when comparing the different levels of education and type of delivery (p=0.507 and 0.366, respectively).

At the end of the questionnaire, the participants were asked about their particular views on the ECV procedure. Of the 133 participants, 100% felt that knowledge about the procedure should reach the major population of pregnant women, 131 (98.50%) opined that they would talk to other pregnant women about it, and 113 (84.96%) said that they would perform the procedure if they had indications for it.

## DISCUSSION

From the study conducted, it was possible to observe that there was a positive evolution in the patient's knowledge of ECV after reading the content made available on the social network Instagram. We concluded that there was an increase of 1.015 in the patient's score before and after reading, confirming the hypothesis that Instagram contributed to the knowledge. In addition, in the final questions of the questionnaire, 133 participants were asked about their particular views on the ECV procedure. Of this sample, 100% felt that knowledge about the procedure should reach the major population of pregnant women, 131 (98.50%) opined that they will talk to other pregnant women about it, and 113 (84.96%) said that they would perform the procedure if they had indications for it.

Despite the positive results, it was possible to note during the questionnaire administration that some situations could influence the results of the survey. The first was the lack of concentration of some patients when it came to reading the content on Instagram. This was due to anxiety about going to the physician and leaving, discomfort due to pregnancy symptoms, or even having small children demanding their attention. In order to reduce the influence of this factor, the pregnant women were contacted before their appointment with the physician so that they would not feel like they were wasting time answering the questionnaire but rather using the time they had to wait for the physician to call them. It is possible that the results would have been even better if the questionnaire had been administered at a time already scheduled and reserved for pregnant women. In addition, while analyzing the results, it was hypothesized that pregnant women who were more likely to need the ECV, who were more interested in it due to personal factors, and who had a higher level of education might have a better outcome than other women. However, this possible relationship was resolved by analyzing the relationship between the outcome and the education level and between the outcome and the desired delivery type. There was no statistically significant difference when comparing the different levels of education or different delivery type preferences.

According to a survey conducted by Comscore, Brazil is the third most social media-consuming country in the world. With a reach of 81.4%, Instagram is the third most accessed social media by Brazilian users^11^. The Internet will reach 90.0% of the country's households by 2021. People aged 25 to 29 years have the highest percentage of social media use, 94.5%, but all age groups between 14 and 49 years have percentages above 90%^12^. These data show how Instagram can be used as a tool for the population to identify their health aspirations and be equipped to deal with their environment^13^. Therefore, when health content is shared on Instagram, as was the case with the ECV, it opens an opportunity for the population to learn about an alternative other than those usually proposed, such as a cesarean section in the case of a breech presentation. In this way, pregnant women can bring this option to their appointment with the obstetrician, opening up a space for discussion about the different options, so that the physician feels the need to update himself on meeting the expectations of his pregnant women, and also so that the woman is an active part of her care process and not just someone who follows orders. There is evidence that engaged pregnant women are more compliant with the treatment process and are better informed to make treatment choices that fit their lifestyle and desired outcomes^14^.

Something very important about Instagram, and social media in general, compared to other ways of promoting health knowledge, is their durability and reach. For example, if pamphlets had been used in the research as a way of disseminating information about ECV, the knowledge would only have reached the patients in our service, but by using Instagram as a method of disseminating knowledge, the reach was increased since the page on this social media is available to anyone looking for information on the subject. In addition, the pregnant women who participated in the research can recommend the page to their friends. For example, many of the pregnant women who responded to the questionnaire were asked to follow the page so that they could read the content at home and show it to their friends. Therefore, social media can widen access to those who do not have easy access to health information through traditional methods, such as younger people, ethnic minorities, and lower socioeconomic groups^15^. And in addition to accessibility, social media are a cheaper way to disseminate information than more traditional methods, such as billboards, commercials, flyers, or posters.

In a study conducted by Rooyen^16^ on the communication of science news through social media in South Africa, the author argues that, during the Ebola outbreak, social media played an extremely important role in the dissemination of science-related news and information. The author concluded that seemingly equal amounts of media coverage were devoted to the positive role that social media played in helping combat the pandemic and the negative role that social media played in enabling the rapid, rampant spread of misinformation about the disease.

## CONCLUSION

This study provided a comprehensive overview of pregnant women's profile and knowledge about ECV, as well as the effectiveness of disseminating information through Instagram. In addition, pregnant women expressed a strong desire to share information about ECV with other pregnant women.
